# Prescribing patterns of glucosamine in an older population: a national cohort study

**DOI:** 10.1186/1472-6882-13-316

**Published:** 2013-11-13

**Authors:** Rose Galvin, Grainne Cousins, Fiona Boland, Nicola Motterlini, Kathleen Bennett, Tom Fahey

**Affiliations:** 1 HRB Centre for Primary Care Research, Department of General Practice, Royal College of Surgeons in Ireland, Dublin, Ireland; 2 School of Pharmacy, Royal College of Surgeons in Ireland, Dublin, Ireland; 3 Department of Pharmacology and Therapeutics, Trinity College Dublin, Dublin, Ireland

**Keywords:** Osteoarthritis, Glucosamine, Cost-effectiveness

## Abstract

**Background:**

Glucosamine is commonly prescribed as a disease modulating agent in osteoarthritis. However, the evidence to date suggests that it has a limited impact on the clinical symptoms of the disease including joint pain, radiological progression, function and quality of life. The aim of this study was to examine the prescribing patterns of glucosamine from 2002–2011 in an elderly Irish national population cohort using data from the Health Service Executive Primary Care Reimbursement (HSE-PCRS) General medical services (GMS) Scheme.

**Methods:**

Patients aged ≥ 70 years on the HSE-PCRS pharmacy claims database between January 2002 and December 2011 were included. ATC code M01AX05 (glucosamine) was extracted. Prevalence rates per 1000 eligible population with 95% confidence intervals were calculated for all years and age groups (70–74 years, ≥75 years). A negative binomial regression analysis was used to determine longitudinal usage trends and compare prevalence rates across years, sex and age groups.

**Results:**

The annual patient rate of glucosamine prescribing increased significantly from 13.0/1000 eligible population (95% CI 12.6-13.4) in 2002 to 68.7/1000 population (95% CI 67.8-69.5) in 2009 before decreasing to 62.4/1000 population (95% CI 61.6-63.2) in 2011. The rate of prescribing of glucosamine varied with sex, with women receiving significantly more prescriptions than men. The cost of glucosamine also increased from 2002–2008. In 2008 total expenditure reached a high of €4.6 million before decreasing to €2.6 million in 2011.

**Conclusion:**

The national trend in prescribing of glucosamine increased significantly from 2002 to 2009 before decreasing in 2010 and 2011, in keeping with current international guidelines. There is a need for awareness among healthcare professionals and patients alike of the best available evidence to inform decision making relating to the prescription and consumption of such supplements.

## Background

Arthritis affects approximately 714,000 people in Ireland, accounting for one in three visits to general practitioners (GPs) [1]. Osteoarthritis (OA) is the most common form of arthritis and is the leading cause of disability in the elderly [2]. The prevalence of OA is expected to increase in the coming years as risk factors, such as an ageing population and obesity become more prevalent [3]. OA accounts for considerable clinical and economic burden as a result of reduced quality of life, increased use of health care resources and loss of productivity [4]. Non-pharmacological therapy is considered to be the foundation for the successful management of OA and consists of individualised patient specific programmes incorporating elements of dietary advice, physiotherapy, exercise and patient education. Pharmacological options include the prescription of analgesics and non steroidal anti-inflammatory drugs to provide pain relief but the adverse cardiovascular and gastrointestinal events associated with long term use have led healthcare professionals to consider other options [5].

For the past number of years, the cartilage constituents such as glucosamine and chondroitin have been used as disease modulating agents [2,3]. Glucosamine is an amino sugar, and an important precursor in the biochemical synthesis of glycosaminoglycans, which are part of the structure of cartilage. Chondroitin also serves as a building block for cartilage [3,6,7]. It is hypothesised that the oral administration of these substances may prevent further damage to joints that have experienced cartilage degeneration. The efficacy and safety of glucosamine and chondroitin alone or in combination versus placebo with respect to clinical symptoms, function, quality of life and ability to modify structural changes of OA has been examined extensively. Three systematic reviews with meta-analysis failed to demonstrate that glucosamine and chondroitin (or a combination of the two) had an impact on progression of OA [3,5,8], in terms of joint pain, radiological progression of the disease, function and quality of life. In 2008, the UK National Institute for Clinical Excellence (NICE) produced guidelines stipulating that ‘the use of glucosamine or chondroitin products is not recommended for the treatment of osteoarthritis’ [9]. These were followed by similar recommendations in clinical guidelines on the treatment of OA of the knee from the American Academy of Orthopaedic Surgeons in 2010 [10]. However, in spite of scientific evidence demonstrating little or no benefit of glucosamine sulphate over placebo, there continues to be a growth in their use with global sales of glucosamine reaching almost €1.47 billion in 2009 [11]. It is anticipated that sales will continue to grow, with a forecasted increase in sales to €1.7 billion in 2013. In the Irish context, cartilage constituents are available as over-the-counter supplements and on prescription. Until recently, glucosamine was available free on prescription to people aged ≥ 70 years as part of the National Shared Services Primary Care Reimbursement Service of the Health Service Executive in Ireland (HSE-PCRS) general medical services (GMS) scheme. Chondroitin is not available for reimbursement under this scheme. The aim of this study is to examine the prescribing patterns of glucosamine over a ten year period from 2002–2011 in an elderly Irish national population using data from the HSE-PCRS pharmacy claims database. A secondary objective of the study is to identify the cost of prescribing of glucosamine to the State.

## Methods

### Study population and data source

This was a national cohort study of patients aged ≥ 70 years between January 2002 and December 2011. The HSE-PCRS general medical services scheme provides free health services including medications to eligible persons in Ireland. The scheme was means tested for those aged <70 years and free for those aged ≥70 years between July 2001 and December 2008 and over 97% of this age group availed of the scheme nationally during this time [12,13]. From January 2009 the Irish government removed the ‘automatic’ entitlement to a free health services for persons ≥ 70 years and there is now a requirement that people ≥ 70 years satisfy a means test to receive free health services. However, approximately 95% of people ≥ 70 years are still entitled to free health services under the revised scheme [14].

The HSE-PCRS pharmacy claims database of dispensed medications was used to identify the study population. The pharmacy claims database provides details on monthly dispensed medications for each individual within the scheme. Prescriptions are coded using the World Health Organization Anatomical Therapeutic Chemical (ATC) classification system [15]. Prescriber information, defined daily doses (DDD), strength, quantity, method and unit of administration of each drug dispensed, net ingredient cost and pharmacist dispensing fee per item dispensed are also available. No information on diagnosis or disease condition is available but prescriptions of glucosamine were used as proxies for diagnosis of OA. This method has been used in previous studies of this nature [12,13].

### Ethical approval

Permission was given by the data controller to use the GMS dataset if anonymised and analysed at group level. Therefore it was unnecessary to seek specific ethical approval.

### Definitions and inclusion criteria

Patients aged ≥ 70 years on the HSE-PCRS database between January 2002 and December 2011 were included in this study. For the purposes of this study, ATC code M01AX05 (glucosamine) was extracted. We defined long term users as those who were prescribed glucosamine for six or more consecutive months during the study period. Short term users were defined as people with a dispensing pattern that did not meet the definition for long term use i.e. less than six consecutive months. These definitions are consistent with those used in a previous large randomised controlled trial that examined the impact of glucosamine and chondroitin in people with OA [16]. A six month history of the prescribing pattern of glucosamine was ascertained individuals who received a glucosamine prescription in January 2002 to establish prescribing history.

### Statistical analysis

Prevalence rates per 1000 GMS population and associated 95% confidence intervals for individuals aged ≥ 70 years were calculated as a proportion of all eligible persons (≥ 70 years) entitled to free health services, as identified from the annual reports produced by the PCRS. Prevalence rates of glucosamine prescribing and 95% confidence intervals were also calculated across years, age groups (70–74 years, ≥75 years) and sex. A negative binomial regression model was used to determine trends in prescribing rates. The log of the GMS population was used as the offset term and year, age group, sex and all possible interactions between these variables were included in the model. Additionally, annual costs were calculated as the total expenditure which includes the net ingredient cost of the drug, value added tax and pharmacist dispensing fee. The data were analysed using Stata version 11 (StataCorp, College Station, Tx, USA) and SAS version 9.3 (SAS Institute Inc. Cary, NC, USA). The Bonferroni method was used to adjust for multiple comparisons and *p-*values <0.05 were deemed significant.

## Results

### Descriptive statistics

From January 2002 to December 2011, the number of individuals aged ≥70 years identified from the Irish HSE-Primary Care Reimbursement Services (PCRS) pharmacy database and the PCRS annual reports that are entitled to free healthcare range from 309,330 and 351,853 with a mean of 334, 249 (SD 15,787) individuals identified per year. On average 58% of the study population are female and 42% are male.

### Prescribing trends of glucosamine

**Figure 1:**
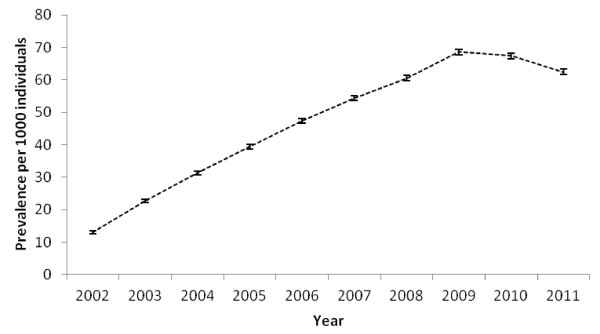
Prevalence rate (per 1000 population) of glucosamine prescribing and 95% confidence intervals (CI) for 2002 -2011.

**Figure 2:**
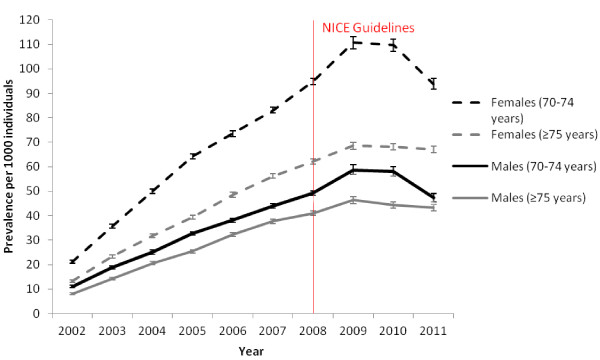
Prevalence rate (per 1000 population) and 95% confidence intervals (CI) of glucosamine prescribing for males and females for two age groups (70-74 years and ≥ 75 years) from 2002 to 2011.

Each year during the study period, between 4,126 and 23,308 people ≥70 years were prescribed glucosamine on at least one occasion. Table 1 displays the prevalence rate of glucosamine prescribing (per 1000 population) over the study period. In 2002, 13.0/1000 population (95% CI 12.6-13.4) received at least one glucosamine prescription. This figure increased to 68.7/1000 population (95% CI 67.8-69.5) in 2009 before decreasing to 62.4/1000 population (61.6-63.2) in 2011. On average 69% of individuals who were prescribed glucosamine between 2002 and 2008 were defined as long term users and this decreased to 65% between 2009 and 2011. Figures 1 and 2 also illustrate the overall prevalence rate trends in prescribing (Figure 1) and trends by age and sex (Figure 2) during the study period.

**Table 1:** Prevalence rate (per 1000 population) of glucosamine prescribing and 95% confidence intervals (CI) for 2002 - 2011

**Year**	**Prevalence rate (95% CI)**
**2002**	13.0 (12.6-13.4)
**2003**	22.7 (22.2-23.3)
**2004**	31.4 (30.8-32.0)
**2005**	39.4 (38.7-40.1)
**2006**	47.4 (46.6-48.1)
**2007**	54.4 (53.6-55.2)
**2008**	60.5 (59.7-61.3)
**2009**	68.7 (67.8-69.5)
**2010**	67.4 (66.5-68.2)
**2011**	62.4 (61.6-63.2)

### Sex and age differences

Table 2 shows the prevalence rates and 95% confidence intervals for both sexes and age groups for each year of the study. The fixed term, sex × age group × year, was significant in the model (*p =* 0.0004) and hence, by the hierarchical principle, all lower order terms involving these variables (sex, age group, year, sex × age group, sex × year and age group × sex) were included in the model. The estimated differences in prevalence of glucosamine between the males and females from the model for both age groups and for all years were calculated. Significant differences in prevalence rates of glucosamine were observed for males and females for both age groups for all years, with one exception. In 2007, there was no significant difference in the prevalence between males in the 70–74 age group and males in the ≥ 75 age group. Females, for both age groups, were significantly more likely to receive a glucosamine prescription than males and additionally males and females in the 70–74 year age group were significantly more likely to receive a glucosamine prescription than in the ≥ 75 age group.

**Table 2:** Prevalence rate and 95% confidence intervals (CI) of glucosamine prescribing for males and females for two age groups (70–74 years and ≥ 75 years) from 2002 to 2011

**Year**	**Prevalence rate (per 1000 population)**			
	**Males (95% CI)**		**Females (95% CI)**	
	**70-74 years**	**≥ 75 years**	**70-74 years**	**≥ 75 years**
**2002**	11.1 (10.5-11.6)	8.0 (7.6-8.4)	21.0 (20.4-21.7)	13.1 (12.6-13.6)
**2003**	18.9 (18.3-19.5)	14.2 (13.7-14.7)	35.8 (35.0-36.6)	23.3 (22.7-24.0)
**2004**	25.1 (24.3-25.9)	20.5 (19.9-21.2)	50.0 (49.0-50.9)	31.9 (31.1-32.6)
**2005**	32.8 (32.0-33.5	25.4 (24.7-26.1)	64.1 (63.1-65.2)	39.5 (38.6-40.3)
**2006**	38.1 (37.2-38.9)	32.3 (31.5-33.1)	73.6 (72.4-74.7)	48.6 (47.6-49.5)
**2007**	43.9 (43.0-44.8)	37.8 (36.9-38.6)	83.1 (81.9-84.3)	56.2 (55.2-57.2)
**2008**	49.1 (48.2-50.1)	41.0 (40.1-41.9)	94.8 (93.5-96.1)	62.2 (61.1-63.2)
**2009**	58.7 (56.8-60.7)	46.3 (45.0-47.7)	110.8 (108.3-113.3)	68.6 (67.2-69.9)
**2010**	58.0 (56.1-60.0)	44.3 (42.9-45.6)	109.7 (107.2-112.2)	68.1 (66.8-69.5)
**2011**	47.3 (45.7-49.0)	43.3 (42.0-44.7)	93.8 (91.6-96.0)	67.2 (65.8-68.5)

### Cost

The cost of glucosamine prescribing during the study period is displayed in Table 3. The net ingredient cost of glucosamine increased from €419,497 in 2002 to almost €4 million in 2008. Costs started to decrease in 2009 and were approximately €1.9 million in 2011. The total expenditure rose from €457,378 in 2002 to over €4.6 million in 2008 before decreasing to just under €2.6 million in 2011.

**Table 3:** Cost of glucosamine prescribing from 2002 to 2011

**Year**	**Net ingredient cost**	**Total expenditure**
	**(€)**	**(€)**
**2002**	419,497	457,378
**2003**	924,454	1,028,179
**2004**	1,511,438	1,697,102
**2005**	2,117,642	2,380,054
**2006**	2,733,698	3,105,582
**2007**	3,425,933	3,915,349
**2008**	3,979,162	4,624,093
**2009**	3,388,832	4,032,809
**2010**	2,462,673	3,198,501
**2011**	1,855,585	2,558,989

## Discussion

### Principal findings

The findings from this national population based study indicate that the overall rate of glucosamine prescribing in individuals ≥ 70 years increased significantly between 2002 to 2009 before decreasing in 2010 and 2011. The decrease in prescribing trends in recent years is in keeping with the 2008 NICE guidelines where the use of glucosamine or chondroitin products are not recommended for the treatment of osteoarthritis [9]. The rate of prescribing of glucosamine varies with sex, with women receiving significantly more prescriptions than men. Our results also indicate that a significant cohort of patients receive glucosamine on a long term basis.

### Results in the context of current literature

The prevalence and pattern of glucosamine consumption is of significance for public health and future health promotion due to the rise in the elderly population, increasing consumer interest in the value of healthy diet and exercise and growth in public awareness of the importance of preventive health [17]. While prescribing trends have reduced in Ireland in the years following the introduction of the NICE guidelines in 2008, our results indicate that over 6% of individuals over 70 years were prescribed glucosamine in 2011. Similar patterns are evident in the UK where prescribing trends have also dropped, yet the net ingredient cost of glucosamine was over £7 million in 2011 [18]. The lag between evidence availability and uptake by clinicians has been examined and findings from a systematic review suggest that there are a number of barriers to guideline uptake in the primary care. A number of reasons cited by GPs’ for not adhering to clinical guidelines included the robustness of evidence supporting the guideline, GPs’ clinical experience at odds with the guideline recommendation, professional responsibility to the patient, practical issues with guideline uptake and guideline format [19]. A combination of these factors may have contributed to the delay in the uptake of the guidelines in the treatment of OA. However, the totality of evidence demonstrates that there are no clinically meaningful effects of chondroitin, glucosamine or their combination on perceived joint pain or joint space narrowing in individuals who have OA [3]. In light of this recent evidence indicating little or no benefit of glucosamine sulphate over placebo in improving symptoms in patients with OA, a report by the Irish National Centre for Pharmacoeconomics in Ireland concluded that ‘glucosamine sulfate (DONA™) is not a cost effective therapy for the treatment of osteoarthritis in the Irish healthcare setting’ [20]. This has led to the suspension of glucosamine from the HSE-PCRS scheme in September 2012, meaning that GPs can no longer prescribe glucosamine for patients at no cost.

Our results demonstrate that women are significantly more likely to receive a prescription of glucosamine than men. This finding concurs with the current literature suggesting that women prefer complementary medicine in both western and eastern countries [21,22]. In addition, women are known to be more commonly affected by OA than men [2]. We also found that men and women aged 70–74 years were significantly more likely to receive a prescription than those aged ≥75 years. These results are broadly in-keeping with a recent Australian study that examined self-reported patterns of glucosamine use and reported that glucosamine use is higher in those aged 60–79 years than those aged ≥80 years [17]. The study also found that women were significantly more likely to use the supplement, consistent with previous research findings.

### Clinical implications

In the international context, glucosamine is one of the most commonly prescribed complementary alternative medicines (CAM) with recent research identifying 20% of the US adult population using glucosamine [23]. In Australia, glucosamine is the most frequently recommended CAM by GPs and community pharmacists, with 85.2% of GPs and 94.7% of Australian community pharmacists recommending glucosamine to patients [17]. Previous studies of consumer decision-making related to CAM have reported that recommendations of trusted individuals such as health care providers and family and friends appear to play a significant role in non-prescription decision-making [24]. A recent study examined the extent to which the level of scientific evidence supporting the efficacy of CAM impacts consumer decision-making in the self-selection of these products [25]. The authors reported that a small number of participants used direct scientific evidence sources in the decision-making process. However, the majority obtained knowledge of scientific evidence through indirect sources including health care professionals and the media. In the primary care setting, GPs, pharmacists and physiotherapists frequently engage with patients in the management of musculoskeletal conditions, including OA. Therefore, it is imperative that these clinicians have the skills to critically evaluate the quality and validity of external scientific evidence to inform decision-making relating to patient care.

### Strengths and weaknesses of the study

The findings of this study are nationally representative for older adults in Ireland. To our knowledge, no other study examining national prescribing trends of cartilage constituents in an older population have been publised to date. Our findings may represent an underestimate of the true prevalence of consumption as cartilage constituents such as glucosamine and chondroitin are available as over the counter supplements. However, it is unlikely that patients bought glucosamine over the counter during the study period when it was possible to obain them for free under the GMS scheme. Finally, we have used a prescription of glucosamine as a proxy for OA as the HSE-PCRS database does not contain information on disease status. While it is unlikely that individuals received this product for another condition, it may be that some high risk patients were prescribed glucosamine as preventive therapy for OA.

### Future research directions

This study highlights that the national trend in prescribing of glucosamine increased significantly from 2002 to 2009 before decreasing in 2010 and 2011, in keeping with current international guidelines. In the broader context of prescribing both pharmacological and non-pharmacological interventions, the nature and content of interactions between patients and heath care professionals regarding the evidence around the effectiveness of treatments need to be considered in greater detail. In addition, there is a need to identify and develop effective knowledge translation strategies to convey scientific evidence to the lay audience in ways they find personally meaningful.

## Conclusion

This national elderly population based study indicates that the trend in prescribing of glucosamine increased significantly between 2002 and 2009 before beginning to decrease in 2010, in line with the current international guidelines. It is important that healthcare professionals and patients alike are aware of the best available evidence to inform decision making relating to the prescription and consumption of glucosamine.

## Competing interests

The authors declare that they have no competing interests.

## Authors’ contributions

All authors were involved in the study conception and design. KB acquired data for analysis and NM and FB performed statistical analysis. RG and GC interpreted the data and drafted the paper. GC, KB and TF, critically revised the draft manuscript. All authors read and approved the final manuscript.

## Pre-publication history

The pre-publication history for this paper can be accessed here:

http://www.biomedcentral.com/1472-6882/13/316/prepub
